# Innovating HTA: a call for capacity building and standardization

**DOI:** 10.1017/S0266462326103456

**Published:** 2026-01-28

**Authors:** Diana M.J. Delnoij, Dalia M. Dawoud, Jamie Elvidge, Zoltan Kaló, Saskia Knies, Bertalan Németh, Wim Goettsch

**Affiliations:** 1 National Health Care Institute, Netherlands; 2 Erasmus School of Health Policy & Management, Erasmus Universiteit Rotterdam, Netherlands; 3 Science, Evidence and Analytics, National Institute for Health and Care Excellence, UK; 4 Health Technology Innovation Laboratory (HTA Lab), National Institute for Health and Care Excellence, UK; 5 Syreon Research Institute, Hungary; 6 Universiteit Utrecht, Netherlands

**Keywords:** health technology assessment, innovation, capacity building, real-world evidence, European collaboration

## Abstract

**Objectives:**

New technologies are being developed in a context of scarcity. Health technology assessment (HTA) aims to support decision makers in providing equitable and affordable access to effective innovations. This study aims to summarize the policy-related findings of a Horizon2020 project on innovating HTA methods and discuss their implications for the governance of HTA in Europe.

**Methods:**

A thematic analysis of policy-oriented papers (*n* = 18) from the Next Generation Health Technology Assessment (HTx) project was carried out to summarize challenges and solutions. Subsequently, via an online survey and in a 2-day meeting, European and global stakeholders (*n* = 21) were invited to comment on these solutions and to prioritize future strategies.

**Results:**

Reported challenges included a lack of access to standardized data, differences in evidentiary needs, existing policy structures, and a lack of capacity and knowledge. Suggested solutions were capacity building, national and international dialogues, standardization, and increased European collaboration. Stakeholders had different expectations with respect to the likely success of these solutions.

**Conclusion:**

Innovation of HTA requires alignment of evidentiary needs through dialogues, standardization through increased European collaboration, and capacity building. However, without additional investments in personnel capacity, HTA agencies must still prioritize some activities at the expense of others. Furthermore, although European collaboration is important, global alignment might be required to enforce standardization.

## Introduction

European healthcare systems face pressure from population ageing and the development of new technologies in a context of financial scarcity and labor shortages. Providing value for money is key to addressing these challenges. Health technology assessment (HTA) aims to contribute to that by supporting informed decision making (1). HTA plays a particularly prominent role with respect to reimbursement decisions on new pharmaceuticals, where HTA agencies perform evaluations that use, at least for the clinical part of the assessment, similar evidence used in submissions by manufacturers to regulatory agencies that decide on market access (2). Therefore, increased alignment between the evidence requirements of regulators and HTA agencies is proposed (3;4).

Largely the same body of clinical evidence that supports reimbursement decisions is also used by guideline developers to formulate recommendations about clinical practice (5). To generate impact, HTA recommendations should lead to measurable changes in clinical practice (6). This implies interdependence between the evidence requirements of HTA agencies and those of clinical practitioners.

In short, HTA is but one step in a whole evidence ecosystem (7). Schünemann et al. (8) argue that decision-making processes of the various actors in this ecosystem are often poorly coordinated. Therefore, the authors call for more agreement on how evidence is being used across the various decision levels in healthcare. Such harmonization is also necessary to offer consistent guidance to the industry on what HTA bodies expect (9).

Between 2006 and 2021, therefore, the European Commission used soft governance instruments to promote coordination and collaboration between European HTA agencies within the European Network for Health Technology Assessment (10). As of 2025, the Health Technology Assessment Regulation (HTAR) requires further alignment of relative effectiveness assessments between European HTA agencies (11).

This alignment remains a challenging issue, because both the health technologies that are being assessed and the state-of-the-art HTA methods are continuously undergoing change and innovation. Therefore, mechanisms need to be in place that guide the process of innovation in HTA methods. Jiu et al. (12) propose the IHTAM Framework (Innovation of HTA Methods), developed within the Horizon2020 Next Generation Health Technology Assessment (HTx) project, to guide the innovation process. The HTx project aimed to support patient-centered, societally oriented, and real-time decision making for integrated healthcare throughout Europe. Based on a number of case studies, methods have been developed for integrating evidence from randomized controlled trials (RCTs) and real-world evidence (RWE) based on real-world data (RWD) (13), as well as for forecasting treatment effects and costs based on artificial intelligence (AI) and machine learning methods.

Within the HTx project, a dedicated work package was aimed at providing policy solutions for implementing these new methods. This included international consensus building between HTA organizations in Europe and between HTA organizations, regulators, and guideline-developing organizations. In addition, a work package focused on the transferability of innovative funding and reimbursement models for use throughout Europe. In this study, we aim to summarize the findings of this policy-oriented work of the HTx project and discuss their implications for the governance of HTA in Europe. Our specific research questions are the following:

1. Which governance challenges for innovation of HTA methods are reported in articles describing the findings of the HTx project?
2. Which policy solutions are suggested in articles describing the findings of the HTx project?
3. How feasible are these solutions according to international stakeholders and experts in the field of HTA, market access, and clinical guideline development?

## Methods

### Data

To answer the first two research questions, we included results from the policy-oriented studies of the HTx project (see Supplementary Appendix 1). Data collection for the third research question took place before and during the final Policy and Expert Forum (hereafter: Forum) meeting of the HTx project, which convened in Sitges on 23–24 May 2024. Participants (*n* = 21) were recruited from a variety of European and global stakeholders, including policy makers, industry, patient organizations, clinicians, and academia. Before the meeting, participants were asked to comment on the expected success of potential solutions and to prioritize future strategies via an online survey (see Supplementary Appendix 2). The results of the online survey were presented and discussed during the Forum meeting. Extensive notes were taken during the discussion.

### Analysis

The findings reported in the policy-related publications of the HTx project have been summarized on a data sheet (Supplementary Appendix 1). The results will be described thematically, focusing on evidence requirements and RWD, stakeholder involvement and alignment throughout the evidence ecosystem, implementation in payment models, and AI and machine learning in relation to HTA. The results from the online survey have been analyzed with descriptive statistics. Relevant issues that emerged from the discussion during the Forum meeting have been summarized.

### Ethics

Informed consent was obtained from the participants in the Forum for using their survey responses and information from the minutes of the meeting.

## Results

### Challenges and solutions reported in articles of the HTx project

#### Evidence requirements and RWD

Using COVID-19 as an example of a situation in which – initially – there was a lack of evidence, Elvidge and Dawoud (Supplementary Appendix 1, no. 5) aimed to understand key challenges in assessing COVID-19 technologies. Based on a round-table discussion, the authors recommended the development of an interim best-practice HTA framework. In a next step, therefore, Elvidge et al. (Supplementary Appendix 1, no. 6) developed such a best-practice guidance using a sandbox approach, previously described by Leckenby et al. (Supplementary Appendix 1, no. 14) as a promising methodology for fostering innovative policies to address known challenges and disruptive health technologies. The guidance codeveloped by Elvidge et al.’s (Supplementary Appendix 1, no. 6) multistakeholder sandbox recognizes the need for pragmatic decision making in the case of rapidly changing disease characteristics, evidence, clinical practice, and pressures on healthcare systems and decision makers. The authors encourage HTA agencies to accept different types of evidence, including RWE, where high-quality RCTs are lacking, and they call for a responsive, “living” HTA approach, in which assessments will be rapidly revisited when new or better evidence becomes available. To illustrate this, Elvidge et al. (Supplementary Appendix 1, no. 7) updated a systematic review summarizing the current cost-effectiveness evidence regarding tests for SARS-CoV-2 and treatments for COVID-19.

Although the use of RWD and RWE is encouraged by Elvidge et al. (Supplementary Appendix 1, no. 6), using these data is not common practice in HTA. Hogervorst et al. (Supplementary Appendix 1, no. 8) assessed which challenges in HTA, related to the increasingly complex nature of new health technologies, make the acceptance of RWD most likely. Examples of complex new technologies are advanced therapy medicinal products (ATMPs), histology-independent treatments, and sequences or pathways of treatments (Supplementary Appendix 1, no. 8). According to Hogervorst et al. (Supplementary Appendix 1, no. 9), European HTA organizations appear to be generally positive toward increased use of RWD, for example, for the assessment of orphan drugs or treatments for subgroups of patients with more common diseases. HTA agencies see patient registries as a potentially useful source. However, many barriers to the use of RWD were also reported, including a lack of RWD sources (Supplementary Appendix 1, no. 8).

Kamusheva et al. (Supplementary Appendix 1, no. 13) aimed to identify the main barriers to the application of RWE derived in Western European countries for the purposes of decision making in healthcare in Central and Eastern European (CEE) countries. Transferability is hampered by the following:

- technical barriers (e.g., lack of expertise or financial resources);
- regulatory barriers (e.g., lack of unified guidance and lack of standards);
- clinical and scientific barriers (e.g., differences in population characteristics and in medical practice);
- perceptional barriers (e.g., uncertainty regarding quality and limited trust in RWE).

Németh et al. (Supplementary Appendix 1, no. 15) deem it implausible that HTA agencies in CEE will have access to patient-level data collected in Western European jurisdictions. Correspondingly, they can only draw conclusions from published, aggregate-level RWE generated elsewhere. The authors, therefore, call for the following:

- a European consensus on mandatory dissemination of RWE reports in a standardized manner;
- joint international training;
- the development of a transferability checklist;
- the development of best practice guidelines;
- the development of an open, transparent database with RWE that can be used for HTA purposes as part of the European HTA Regulation;
- adherence to the principles that research output should findable, accessible, interoperable, and reusable.

#### Stakeholder involvement and alignment throughout the evidence ecosystem

The relationship between HTA and clinical guideline development was a specific research topic of the HTx project (Supplementary Appendix 1, no. 16), as was the topic of patient involvement.

Hogervorst et al. (Supplementary Appendix 1, no. 10) aimed to quantify the similarities and discrepancies between European HTA reports and clinical guidelines for multiple sclerosis. Final recommendations appear to be similar in 90 percent of the cases. However, there are considerable differences in treatment lines and subindications. The authors observe a lack of systematic consultations between HTA organizations and clinical guideline developers, as well as time lags between the publication of HTA reports and subsequent updates of clinical guidelines. The authors recommend more stakeholder dialogue. In a follow-up publication, Hogervorst et al. (Supplementary Appendix 1, no. 10) describe how convergence of evidentiary needs among stakeholders may be achieved, namely, through the following:

- improved communication via multistakeholder early dialogues;
- shared definitions (e.g., of patient populations);
- standardized outcome measures;
- shared methods.

Patients’ perspectives in HTA have been studied specifically in CEE countries. Dimitrova et al. (Supplementary Appendix 1, no. 4) set out to map potential barriers to patient involvement in HTA in CEE countries. A total of twenty-five potential barriers were identified, such as a lack of defined rules on how and when to include patient representatives, a lack of sufficiently explained methodology for the patient’s role in the HTA process, patients’ lack knowledge in regard to HTA and regulatory processes, and use of medical language. Jakab et al. (Supplementary Appendix 1, no. 12) formed recommendations to overcome these critical barriers, for example, training and education, revised procedures, fair compensation, and funding. Dimitrova et al. (Supplementary Appendix 1, no. 4) argue that the development of CEE-specific guidelines can build on general guidelines for patient involvement in HTA, for example, those developed by the European Patients Academy on Therapeutic Innovation or by the Health Technology Assessment international Interest Group for Patient and Citizen Involvement in HTA.

#### Implementation in payment models

The use of innovative payment models was another specific HTx topic, with a focus on implementation of these models in CEE countries. Hogervorst et al. (Supplementary Appendix 1, no. 9) suggest spreading financial risks in relation to outcome uncertainties through pricing and reimbursement schemes. Callenbach et al. (Supplementary Appendix 1, no. 3) studied the actual implementation of innovative payment and reimbursement models in CEE and Middle Eastern (ME) countries. Although financed-based reimbursement models (e.g., discounts and rebates) are widely used, the majority of stakeholders in CEE and ME countries indicated that they would prefer to use outcome-based reimbursement models more often in the future. However, several barriers prevent the widespread use of outcome-based models, such as the following:

- information technology and data infrastructure;
- transaction costs and administrative burden;
- measurement issues;
- governance and regulatory frameworks.

Callenbach et al. (Supplementary Appendix 1, no. 3) recommend dialogues at national and international levels, more European collaboration and international initiatives, and the use of pilot studies to overcome these barriers.

In their study of potential barriers to the use of delayed payment models related to health technologies with high upfront costs in CEE countries, Ádám et al. (Supplementary Appendix 1, no. 1) point to similar barriers, such as transaction costs and administrative burden of delayed payment models, measurement problems, lack of information technology and data infrastructure, governance issues, and perverse policy outcomes. Their practical recommendations to address these barriers include the following:

- using contract archetypes;
- linkage of databases, reusing existing data, and using pilots;
- changes in the European and national accounting rules to allow accruals over several years;
- strengthened HTA system to promote value for money and affordability concepts;
- joint procurement by smaller countries to increase purchasing power.

Ádám et al. (Supplementary Appendix 1, no. 2) provide additional recommendations to facilitate outcome-based reimbursement models, such as the following:

- capacity building in health economics and outcomes research;
- additional data collection to validate surrogate outcomes;
- linkage of databases and reusing existing data;
- use of pilots.

#### AI and machine learning in relation to HTA

Case studies in HTx were aimed at using AI and machine learning in prediction modeling. However, these innovative techniques may be difficult to implement in HTA processes. Tachkov et al. (Supplementary Appendix 1, no. 17) and Zemplényi et al. (Supplementary Appendix 1, no. 19) studied which barriers play a role in CEE countries and formulated recommendations:

- Human factors-related barriers could be remedied by focusing on educating HTA doers and users, establishing collaborations and sharing best practices.
- Regulatory and policy-related barriers could be addressed by increasing awareness and political commitment and by improving the management of sensitive information for AI use.
- Data-related barriers call for enhanced standardization, collaboration with data networks, improved management of missing and unstructured data, application of analytical and statistical approaches to address bias, use of quality assessment tools and quality standards, improved reporting, and creation of better conditions for the use of data.
- Technological barriers could be overcome through sustainable development of AI infrastructure.

### Feasibility of proposed solutions according to stakeholders and experts

The online survey was completed by fourteen members of the Forum before the meeting: five representatives of research institutes or academia, two representing national or international governmental bodies, two representing HTA bodies, two representing industry, two representing clinicians, one representing patient organizations, one representing payers, and two representing other stakeholders.

**Figure 1.**
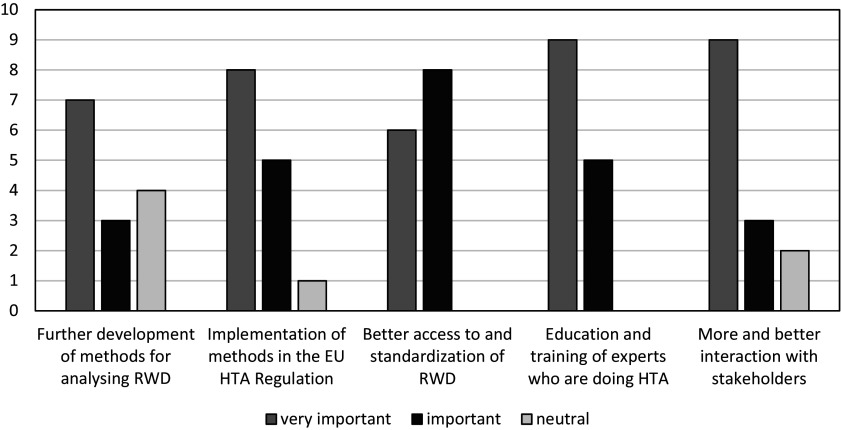
Importance of suggested solutions for further implementation of innovative HTA methodology according to participants of the HTx Policy and Expert Forum (*n* = 14). HTA, health technology assessment; HTx, Next Generation Health Technology Assessment; RWD, real-world data.

First, respondents were asked to rate the importance of suggested solutions in the HTx studies on a scale from 1 (very unimportant) to 5 (very important). Figure 1 shows a graphic representation of the frequency of the responses.

None of the suggested solutions was regarded as unimportant. However, educating and training HTA doers in applying new methods came out as the most important solution for enhancing the uptake of those methods, whereas a focus on further refining the methods for analyzing RWD was seen as the least important. During the meeting, stakeholders discussed the issue of capacity building. Education and training were seen as relevant, but HTA representatives pointed out that, in addition, the work force was an important issue. First experiences with reassessments using RWD, for example, showed that these are labor-intensive and time-consuming.

In addition, respondents were asked whether the proposed solutions were likely to result in more frequent use of new HTA methods that include the integration of RWE for complex health technologies in Europe in the next 5 years. They were asked to estimate the likelihood of that for capacity-building programs and for the introduction of the European Health Data Space (EHDS). It was deemed as likely to be successful by, respectively, 64 percent and 57 percent of the respondents. Furthermore, they were asked to estimate the likelihood of European HTA under the EU HTAR being able to enforce the use of standardized sets of outcome measures that include generic measures of quality of life. Half of the respondents thought that this was (very) likely, but 29 percent thought this was (very) unlikely. Participants in the Forum generally acknowledged the lack of standardization of RWD as a problem. However, for the future, some hope was being placed in AI solutions to create structured data from unstructured sources.

Regarding stakeholder involvement, respondents were asked to choose the three stakeholders they would spend most resources on if they were in charge of an HTA agency and had to prioritize for reasons of efficiency. Respondents prioritized collaboration with regulators (*n* = 10), patient organizations (*n* = 9), and other European HTA agencies (*n* = 8). Only one respondent would prioritize collaboration with non-European HTA agencies. Forum participants stressed the importance of involving clinicians in future HTA methods development, because clinicians also rely on evidence synthesis methods when developing guidelines.

## Discussion

The HTx project was initiated to support patient-centered, societally oriented, and real-time decision making for integrated healthcare throughout Europe. Decision makers are confronted with disruptive new technologies, such as ATMPs or histology-independent treatments, for which current methods of assessment are not always adequate. In addition, the public demands access to interventions that address previously unmet needs, for example, in the field of orphan drugs. These interventions are targeted on small populations that are difficult to tackle with regular HTA methods as well. Overall, the level of scientific evidence of treatment effect in new applications of reimbursement has been decreasing with time (14). Against this background, the aim of the HTx project was to contribute to future-proofing HTA by innovating HTA methods.

Our first research question was related to the governance challenges for the innovation of HTA methods that are reported in the policy-oriented work of HTx. The results show that a large variety of human-factor, technological, and methodological problems complicate the use of RWD and of AI-driven modeling. In terms of policy and governance challenges, several issues resonate throughout the various HTx studies. These challenges include limited access to and lack of standardization of RWD and RWE; a lack of alignment of evidentiary needs throughout the whole ecosystem of regulation, reimbursement and clinical use, existing policy structures that complicate the use of RWE, and a lack of capacity and knowledge, not only on how to use innovative data-driven methods but also on how to genuinely make the most of patient involvement.

In a recent systematic review on the use of RWD in HTA, Zisis et al. (15) report similar challenges, for example, with data access and data quality, and a lack of expertise among stakeholders to critically evaluate evidence from RWD in decision making. In addition, the authors point to the prevailing preferences for clinical trials and systematic reviews, which they label as “a traditional approach to evidence” (15, p. 8). Claire et al. (16) used a series of stakeholder workshops on RWE in HTA, summarizing issues like limited trust in RWE, concerns about data quality, and uncertainty about best practices. A recent study suggests that across European jurisdictions, the representativeness of the data source, study transparency, and the use of robust methodologies are important criteria for RWE acceptance (17). Overcoming the reported challenges is important. RWD offer opportunities to capture evidence on treatment sequence effectiveness, or they can serve as external control arms (18–20). Graili et al. (21) claim that RWE holds the potential to reduce uncertainty and accelerate reimbursement decisions but also acknowledge that the implementation of RWE in HTA is a complex process.

In several of the publications summarized in this study, the challenges were analyzed specifically for CEE countries. However, as was pointed out by Dimitrova et al. (Supplementary Appendix 1, no. 4), the majority of difficulties with patient involvement in HTA in CEE countries could be generalizable for countries with more advanced HTA systems (cf. 22;23). The same is probably true for the challenges reported by Zemplényi et al. (Supplementary Appendix 1, no. 18), who studied barriers that actors in CEE countries encounter when using AI-driven evidence in HTA, and for the problems with innovative payment models reported by Ádám et al. (Supplementary Appendix 1, no. 1). Such payment models are implemented to cope with uncertainty but pose new tensions for existing procedures and for relations with important stakeholders such as patients and clinicians without whom data collection is impossible (24). In Western European countries, issues such as data infrastructure, transaction costs, and administrative burden also play a role when implementing delayed, outcome-based payment models (Supplementary Appendix 1, no. 3). CEE countries lag behind in HTA capacities (25). Their experiences, however, can accentuate structural problems that may also occur in Western European countries. The experiences in the HTx project, therefore, show how valuable the inclusion of studies on transferability is for highlighting the most pressing obstacles to future-proof HTA. Addressing these obstacles on a European level will improve the situation not only in CEE countries but also in Europe as a whole.

The second research question addressed in our study, therefore, was the following: Which policy solutions are suggested in articles describing findings of the HTx project? The HTx studies describe solutions in the realms of capacity building through education and training, and the sharing of best practices; standardization and alignment of data definitions, outcome measures, and reporting formats; national and international dialogues; and increased European collaboration. Regarding the latter, Németh et al. (Supplementary Appendix 1, no. 15) call for an open, transparent database with RWE that can be used for HTA purposes as part of the European HTA Regulation. Other authors have proposed similar solutions. According to Claire et al. (16), facilitators to enhance the use of RWE in HTA consist of multidisciplinary training and education and of stakeholder collaboration.

The need for capacity building within HTA agencies is currently addressed, for example, through tenders of the 2023 EU4Health Work Programme. In addition, the EU-funded SUSTAIN-HTA project (Support Utilization of Sustainable and Tailored Innovative Methods for HTA) will support the implementation of innovative HTA methods in the HTA community and, by extension, support the methodology subgroup of the Coordination Group on Health Technology Assessment in implementing the EU HTAR. This methodological support will be beneficial, particularly given the fact that the EU HTAR applies to products that HTA agencies label as complex and difficult to assess (Supplementary Appendix 1, no. 9). However, as was pointed out by Forum participants, capacity building entails more than training. Without additional investment in personnel capacity, HTA agencies may have to prioritize, for instance, whether to focus on initial assessments or on implementing a lifecycle approach with reassessments based on RWD. Unfortunately, during the Forum, there was scant opportunity to dive into the underlying causes of this lack of trained experts.

To enhance access to standardized RWD, high hopes are placed on the EHDS, although much depends on its actual implementation (26). However, the usefulness of RWD for HTA does not only depend on issues of access and interoperability. It is of vital importance, for example, that standardized patient-relevant outcomes are recorded, including measures of health-related quality of life (4). An open question, however, is who could impose such a standard. Forum participants had different opinions on the opportunities that the EU HTAR could play in standardization of outcome measures. From that perspective, some form of global alignment of evidence requirements may be useful, even though Forum participants did not prioritize collaboration of European HTA agencies with agencies outside Europe.

To explore the practical implications of the various proposals, our third research question was the following: How feasible are these solutions according to international stakeholders and experts in the field of HTA, market access, and clinical guideline development? Forum participants had different expectations with respect to the likely success of the proposed solutions. It was highlighted that performing and evaluating RWD studies will require not only training but also extra personnel. If this is not available, there might be a need for reallocation of resources within the agencies with a risk that other tasks might be less prioritized and that there will be insufficient resources for capacity building.

### Limitations

Our findings are primarily based on studies conducted within the scope of the HTx project. However, we thoroughly discussed our conclusions with participants of the Forum. This Forum consists of senior representatives from the most important stakeholders in HTA and representatives from other relevant European scientific projects and scientific societies.

### Conclusion

Innovation of HTA methodology is challenged by a lack of access to standardized RWD and RWE, existing policy structures that complicate the use of RWE, and a lack of capacity and knowledge. In addition, there is a need for the alignment of evidentiary needs throughout the whole ecosystem of regulation, reimbursement, and clinical use. Proposed solutions to these challenges are in capacity building through education and training and sharing of best practices; standardization; national and international dialogues; and increased European collaboration. However, although training and education of HTA practitioners will help in implementing innovative methods without additional investments in personnel capacity, HTA agencies must choose, for example, how much time they will spend on stakeholder involvement or whether to focus on initial assessments or reassessments. In addition, although European collaboration may help to divide the work through joint assessments, European HTA agencies still might lack the power to effectively impose certain standards with respect to evidence requirements. Global alignment of these standards, therefore, remains important.

## Supporting information

10.1017/S0266462326103456.sm001 Delnoij et al. supplementary material 1 Delnoij et al. supplementary material

10.1017/S0266462326103456.sm002 Delnoij et al. supplementary material 2 Delnoij et al. supplementary material

## Supplementary material

The supplementary material for this article can be found at http://doi.org/10.1017/S0266462326103456.
