# Supplementary material for: Innovating HTA: a call for capacity building and standardization
Source: Int J Technol Assess Health Care. 2026 Jan 28;42(1):e8. doi: 10.1017/S0266462326103456 (PMC12902166; doi:10.1017/S0266462326103456)
Supplement: Delnoij et al. supplementary material 1 — Delnoij et al. supplementary material [file S0266462326103456sup001.pdf]

No.	Reference	Aim	Data collection	Results & conclusions
1	Ádám I, Callenbach M, Németh B, Vreman RA, Tollin C, Pontén J, et al. (2022). Outcome-based reimbursement in Central-Eastern Europe and Middle-East. Front Med (Lausanne). 2022;9:940886	Outcome-based agreements link payments to health benefits that patients realize from using a novel health technology and include pay-for outcome, conditional treatment continuation, and coverage with evidence development. The aim was to explore the transferability of outcome-based payment methods within and outside the European Union with a special focus on countries in CEE and the ME; to highlight the most important barriers and to recommend potential solutions.	A survey on the use of outcome-based agreements and other payment models (see Appendix 1, no.3); a targeted review of scientific and gray literature, iterative rounds of discussions with HTx consortium members representing different stakeholders in HTA; a review of draft barriers and recommendations with representatives of health care payers and with health care financing experts from CEE and the ME during a policy workshop (n=30 participants).	Barriers occur in five broad areas: (1) transaction costs and administrative burden, (2) measurement issues, (3) information technology and data infrastructure, (4) governance, and (5) perverse policy outcomes. Practical recommendations to address these barriers include a wide range of suggestions such as using contract archetypes, capacity building in health economics and outcomes research, additional data collection to validate surrogate outcomes, linkage of databases and reusing existing data, using pilots, increased transparency around key components, or joint procurement.
2	Ádám I, Callenbach M, Németh B, Vreman RA, Pontén J, Strbad T, et al. Delayed payment schemes in Central-Eastern Europe and Middle-East. Front Med (Lausanne). 2022;9:940371	To list potential barriers for implementing delayed payment models and recommendations on how to address these barriers in lower income countries in CEE an the ME.	Survey, exploratory literature review and iterative brainstorming. A draft list of recommendations was validated in a virtual workshop with payer experts from the two regions. See Áppendix 1, no. 1.	Barriers occur in the areas of (1) transaction costs and administrative burden, (2) payment schedule, (3) information technology and data infrastructure, and (4) governance. Fifteen practical recommendations to address these barriers were formulated, ranging from the use of contract archetypes, adjusting data infrastructures, the use of pilot phases or pilot cases, joint procurement, and the preparation of regulatory legal frameworks.
3	Callenbach MHE, Vreman RA, Mantel-Teeuwisse AK, Goettsch WG. When Reality Does Not Meet Expectations-Experiences and Perceived Attitudes of Dutch Stakeholders Regarding Payment and Reimbursement Models for High-Priced Hospital Drugs. Int J Environ Res Public Health. 2022;20(1):340	To gain an understanding of the implementation of innovative payment and reimbursement models in CEE and countries in the ME and to provide future directions that might be followed.	Survey of payers from CEE and ME countries regarding current use of, future preferences for and perceived barriers with innovative reimbursement and payment models (n=27 payers from 11 countries).	Financed-based reimbursement models (e.g. discounts and rebates) are used more often than outcome-based models. 60% of the respondents indicated that the latter were never used. Upfront payment is the most applied payment model. More than half of the respondents indicated that delayed payment models were rarely ever applied. The majority of stakeholders indicated that they would prefer outcome-based reimbursement models to be applied more often in the future (5 years from now). Similarly, almost 80% of stakeholders indicated that they would like payments at outcomes achieved to be applied more often. Barriers mentioned by stakeholders related to IT and data infrastructure, transaction costs and administrative burden, measurement issues, and governance. Recommendations include dialogues at the national and international levels; more European collaboration and joining international initiatives; and the use of pilot studies.

4	Dimitrova M, Jakab I, Mitkova Z, Kamusheva M, Tachkov K, Nemeth B, et al. Potential Barriers of Patient Involvement in Health Technology Assessment in Central and Eastern European Countries. Front Public Health. 2022;10:922708	There are two approaches to incorporating the patients' voice in HTA, preferably used in a mix: (1) patients, caregivers and/or their representatives directly participate at discussions in different stages of the HTA process, often at the same table with other stakeholders; (2) patient involvement activities can be supported by evidence on patient value and experience collected directly from patients, caregivers and/or their representatives. The aim was to map potential barriers of patient involvement in HTA in countries in CEE.	Scoping literature review (n=34 articles) plus workshop with relevant stakeholders from CEE countries and experts from the HTx consortium to identify additional barriers. An iterative process ran throughout these phases by CEE researchers from the HTx consortium deduplicating, merging and categorizing identified barriers.	A total of 25 potential barriers were identified. Barriers from the perspective of payers/HTA bodies are related to the lack of defined rules how and when to include patients' representatives. From patients' perspective barriers are associated with lack of sufficiently explained methodology for the patient's role in the HTA process, lack of HTA and regulatory processes knowledge, use of medical language etc. Difficulties identified are often not specific for patient engagement in HTA nor for CEE countries, but emerge in patient involvement in healthcare decision-making in general and worldwide. The development of CEE specific guidelines can build on general guidelines on patient involvement in HTA, e.g. those developed by EUPATI or by the HTAi PCIG.
5	Elvidge J, Dawoud D. Assessing Technologies for COVID-19: What are the Challenges for Health Technology Assessment Agencies? Findings From a Survey and Roundtable Workshop. Pharmacoeconomics. 2021;39(12):1455-1463	To understand key challenges in assessing technologies for COVID-19 from the perspective of HTA agencies, and to identify whether there is a case for developing innovative methods to inform COVID-19 reimbursement decisions.	Mixed methods, namely an online survey (n=21 HTA agencies, response rate 45%) and a virtual roundtable (n=11 of 21 HTA agencies) aimed at elaborating on survey findings and reaching consensus on most important challenges.	Challenges were related to methodological issues regarding the assessment of clinical effectiveness and cost effectiveness, and to practical, political issues, and decision making issues. Two additional themes were derived from the roundtable: how HTA agencies had responded to the pandemic so far, and how their role might change over time. It was concluded that an interim best-practice HTA framework to address the key challenges would be valuable.
6	Elvidge J, Summerfield A, Knies S, Németh B, Kaló Z, Goettsch W, et al. Health technology assessment of tests for SARS-CoV-2 and treatments for COVID-19: A proposed approach and best-practice recommendations. Int J Technol Assess Health Care. 2023;39(1):e24	To develop best-practice guidance for health technology assessment (HTA) agencies when appraising diagnostic tests for SARS-CoV-2 and treatments for COVID-19.	Multi-stakeholder workshop of participants (n=21) representing HTA agencies, clinical and patient experts, academia, industry, and a payer, from across Europe and North America; supported by targeted reviews of existing COVID-related methods guidance, engagement with clinical experts, a survey and workshop of HTA agencies, a systematic review of published economic evaluations, and a workshop of health economic modelers.	HTA agencies should consider using other types of evidence, including real world evidence, where high-quality randomized controlled trials are lacking. A "living" HTA approach may be useful, given that this is an evolving disease, with evolving scientific understanding and evidence base. Such a "living" HTA approach would allow for decisions to be efficiently revisited in response to new information. This would particularly be helpful if it were supported by a common "disease model" for COVID-19. The authors recommend innovative ways of engaging with the public and clinicians (suchs a digital and online communication tools), and early engagement with regulators and payers.

7	Elvidge J, Hopkin G, Narayanan N, Nicholls D, Dawoud D. Diagnostics and treatments of COVID-19: two-year update to a living systematic review of economic evaluations. Front Pharmacol. 2023;14:1291164	To update a systematic review to provide an up-to-date summary of the cost-effectiveness evidence regarding tests for SARS-CoV-2 and treatments for COVID-19.	Systematic review.	A large number of economic evaluations of interventions for COVID-19 have been published since July 2021 that can help decision makers prioritize between competing interventions. Remaining evidence gaps include head-to-head analyses, disease-specific utility values, and consideration of different disease variants.
8	Hogervorst MA, Pontén J, Vreman RA, Mantel-Teeuwisse AK, Goettsch WG. Real World Data in Health Technology Assessment of Complex Health Technologies. Front Pharmacol. 2022 Feb 10;13:837302	To assess which challenges in HTA, related to the increasingly complex nature of new health technologies, make the acceptance of RWD most likely, based on practical experiences by European HTA organizations.	Survey of HTA organisations (n=22, response rate 67%).	Assessors from European HTA organizations were positive toward increased use of Real World Data and saw patient registries as potentially useful source, because as a large share of organizations already accept these data. The assessment of orphan drugs or other treatments with small patient populations was regarded as a situation in which RWD were most likely to be accepted. However, many barriers to the use of RWD were also reported, such as a lack of RWD sources and existing policy structures or information governance.
9	Hogervorst MA, Vreman RA, Mantel-Teeuwisse AK, Goettsch WG. Reported Challenges in Health Technology Assessment of Complex Health Technologies. Value Health. 2022;25(6):992-1001	To identify which health technologies are perceived as complex and which aspects of HTA are considered most challenging, to assess the main arguments for perceiving health technologies as complex and HTAs as challenging, and to find the most pressing gaps that can be filled with the development of future-proof methods for HTAs of complex health technologies.	Survey of HTA organisations (n=22, response rate 67%).	ATMPs, histology-independent treatments and sequences or pathways of treatments were considered most complex. Challenging factors were methodological issues, but also data insufficiencies. The results underpin the importance of the methods development, such as evidence synthesis methods, within the HTx project. Other potential solutions are improving data availability and quality, and in spreading financial risks because of outcome uncertainties through pricing and reimbursement schemes.

10	Hogervorst MA, Vreman RA, Zawada A, Zielińska M, Dawoud DM, de Jong BA, et al. Synergy between health technology assessments and clinical guidelines for multiple sclerosis. Clin Transl Sci. 2023;16(5):835-849	To assess similarities and discrepancies between HTA reports as compared to clinical guideines for multiple sclerosis medicines.	Content analysis of 132 HTA reports and 9 clinical guidelines for 16 multiple sclerosis treatments.	Final recommendations for reimbursement and inclusion in clinical guidelines similar in 90% of the cases. However, there are considerable differences in treatment lines and subindications. 42% of HTA reports refer to clinical guidelines; 43% to consultations with clinicians. Six of nine clinical guidelines referred to HTA reports; two referred to HTA consultations. Four out of nine clinical guidelines referenced pharmacoeconomic studies. There seemed to be a lack of systematic consultations between HTA organizations and clinical guideline developers, and time lags were observed between the publication of HTA reports and updates of clinical guidelines.Not all new HTA recommendations for multiple sclerosis treatments had been included in clinical guidelines (yet). More stakeholder dialogue and/or consultation of each other's publications is recommended.
11	Hogervorst MA, Møllebæk M, Vreman RA, Lu TA, Wang J, De Bruin ML, et al. Perspectives on how to build bridges between regulation, health technology assessment and clinical guideline development: a qualitative focus group study with European experts. BMJ Open. 2023;13(8):e072309	To assess how convergence of evidentiary needs among stakeholders may be achieved and to determine to what extent convergence can be achieved.	Qualitative study using eight online dual-moderator focus groups (n=42 participants).	Convergence can be enhanced through improved communication via multistakeholder early dialogues, shared definitions (e.g. to consistently define patient populations, or using standardized outcome measures such as those developed by the International Consortium of Health Outcome Measurement) and shared methods. It is argued that required data sets should be inclusive rather than aligned and to keep deliberation and decision-making processes independent. Results show that alignment for pragmatic clinical trial designs and patient registries is needed and that smaller and lower-income countries should be included alignment efforts. It is concluded that alignment and independence should be carefully balanced. Priority in alignment should be given to treatments most critical to patients with high decision-making uncertainty.

12	Jakab I, Dimitrova M, Houÿez F, Bereczky T, Fövényes M, Maravic Z, et al. Recommendations for patient involvement in health technology assessment in Central and Eastern European countries. Front Public Health. 2023;11:1176200	To form recommendations for critical barriers to patient involvement in HTA in CEE countries	Survey of CEE stakeholders (n=105) plus a face-to-face workshop with CEE and HTA experts (n=36)	Twelve recommendations: (1) Educate HTA/payer organisations on the value and good practices of patient involvement, (2) Acknowledge patients as experts on their condition, similar to health care professionals; differentiate but equally value the input of individual patients, patient representatives, and accredited patient experts; (3) Revise local HTA guidelines and procedures; (4) Nominate a dedicated person/team to be responsible for patient involvement activities with sufficient available capacities at each relevant HTA and decision-making body; (5) Set a certain percentage of the HTA annual budget to be spent on patient involvement as a goal; (6) Fair compensation for time and transportation should be provided for the patients involved in the HTA process; (7) EU-funded calls for the implementation of patient-centric evaluation of health technologies especially in countries with limited experience in patient involvement; (8) Set up an open call for individual patients or patient organisations to register for involvement into HTA; have and implement a clear policy on conflict of interests; (9) Provide tailored training(s) and training materials for patients on HTA and local health policy decision-making procedures; set up a working group of organisations with extensive experience in education and working with patients to act as centre of training of patient experts; (10) Educate patient organisations on collecting data and interpreting scientific evidence based on international educational resources; (11) Patient organisations to aim for a diversified portfolio of funders and to declare funding sources publicly; (12) Normative state funding for NGOs with close auditing and detailed expectations from and responsibilities of patient organisations; neither public, nor private funding should be banned by legislation

13	Kamusheva M, Németh B, Zemplényi A, Kaló Z, Elvidge J, Dimitrova M, Pontén J, Tachkov K, Mitkova Z. Using real-world evidence in healthcare from Western to Central and Eastern Europe: a review of existing barriers. J Comp Eff Res. 2022 Aug;11(12):905-913	To identify the main barriers for application of Real World Evidence (RWE) derived in Western European countries for the purposes ofdecision-making in healthcare inCEE countries.	Scoping literature review, discussions with experts, online workshop with participants (n=57) from CEE (n=12).	Transferability is hampered by technical (e.g. lack of expertise or financial resources), regulatory (e.g. lack of unified guidance and lack of standards), clinical & scientific (e.g. differences in population characteristics and in medical practice), and perceptional barriers (e.g. uncertainty regarding quality, limited trust in RWE).
14	Leckenby E, Dawoud D, Bouvy J, Jónsson P. The Sandbox Approach and its Potential for Use in Health Technology Assessment: A Literature Review. Appl Health Econ Health Policy. 2021 Nov;19(6):857-869	To describe the extent of use of sandboxes in healthcare and assess the potential for the sandbox approach to be used to test and develop emerging HTA methods, policies and processes for innovative technologies.	Systematic review.	The use of regulatory sandboxes in healthcare is relatively new and experiences are limited to the adoption of new technologies, particularly digital health, in high income countrie. HTA agencies should consider a sandbox approach to facilitate the development of policies, methods and processes for innovative and disruptive health technologies. The transferability of this approach to low- and middle-income countries should be assessed.

15	Németh B, Kamusheva M, Mitkova Z, Petykó ZI, Zemplényi A, Dimitrova M, et al. Guidance on using real-world evidence from Western Europe in Central and Eastern European health policy decision making. J Comp Eff Res. 2023;12(4):e220157	To propose solutions to overcome barriers preventing CEE countries from using RWE generated in Western Europe.	Scoping review, a webinar with 57 stakeholders from 12 different CEE countries, a survey of CEE stakeholders (n=69), and a workshop with CEE stakeholders (n=14 participants).	The authors deem it implausible that HTA agencies in CEE will have access to data collected in Western European jurisdictions. Therefore they can only draw conclusions from published RWE generated elsewhere, without being able to analyze underlying patient-level data. Nine most important barriers for using that RWE were identified: Lack of (1) favorable local/national governance frameworks, (2) guidance documents for all EU countries on how to publish and share RWE, (3) cooperation standards and data integration, (4) requirements on how and when to use RWE. (5) uncertainty of the relevance of RWE due to a lack of access to underlying data. (6) Differences in medical practice for specific patient groups, (7) lack of available financial resources for using RWE, (8) uncertainty in the quality of RWE, and (9) differences in predefined criteria for evaluation of the effectiveness of medicines. Multiple solutions were proposed, e.g. the need for a European consensus a on the mandatory dissemination and minimal structural and content standard requirements of RWE publication, either in reports of HTA agencies or in scientific journals; joint international training for different stakeholders; the development of a checklist for the transferability of RWE, of best practice guidelines, and of an open, transparent database with RWE that can be used for HTA purposes as part of the European HTA Regulation; and adherence the FAIR principles.
16	Ofori-Asenso R, Hallgreen CE, De Bruin ML. Improving Interactions Between Health Technology Assessment Bodies and Regulatory Agencies: A Systematic Review and Cross-Sectional Survey on Processes, Progress, Outcomes, and Challenges. Front Med (Lausanne). 2020 Oct 16;7:582634	To synthesis the literature on opportunities and outcomes of interaction and harmonization initiatives between HTA bodies and regulatory agencies.	A systematic literature review supplemented by a cross-sectional survey among European HTA bodies (n=22, response rate 61%) and regulatory agencies (n=6, response rate 18%).	There is a clear need to align evidentiary requirements for HTA bodies and regulatory agencies. Although data needs for HTA bodies and regulatory agencies show differences, there is considerable scope for improved alignment, e.g. through early tripartite dialogues, parallel submission (review), adaptive pathways to licensing, and postauthorization data generation.

17	Tachkov K, Zemplenyi A, Kamusheva M, Dimitrova M, Siirtola P, Pontén J, et al. Barriers to Use Artificial Intelligence Methodologies in Health Technology Assessment in Central and East European Countries. Front Public Health. 2022;10:921226	To identify the barriers that are specifically relevant to the use of AI-based evidence HTA systems in CEE.	Scoping literature review based on n=38 articles plus iterative focus group meetings with HTx team members and a consultation of experts (n=67) recruited from HTx team members' networks.	Studies focusing on the AI usage for HTA decision making are scarce. AI methods described were Natural Language Processing, Data Mining and Machine Learning / Deep Learning. From the available studies potential barriers were identified and classified as data related (e.g. missing data or systematic bias), methodological (e.g. lack of transparency), technological (e.g. lack of capacity to build or maintain IT infrastructure), regulatory and policy related (e.g. compliance issues related to high volumes of sensitive data, or lack of access to patient-level data because of data protection regulation), and human factor related (e.g. lack of knowledge in data governance, or lack of skills and expertise). The HTA community should explore best practices for relying on AI-based evidence in initial assessments and for re-evaluating technologies.
18	Zemplényi A, Tachkov K, Balkanyi L, Németh B, Petykó ZI, Petrova G et al. Recommendations to overcome barriers to the use of artificial intelligence-driven evidence in health technology assessment. Front Public Health. 2023;11:1088121	To make recommendations to support healthcare decision-makers in integrating Arificial Intelligence (AI) into the HTA processes. Barriers addressed are particularly focusing on CEE countries.	Survey of HTA experts from CEE countries (n=77) plus a workshop with wider group of experts (n=23), including HTA and reimbursement decision-makers from both CEE countries and Western European countries.	Recommendations have been developed to address:(1) human factor-related barriers (focusing on educating HTA doers and users, establishing collaborations and best practice sharing); (2) regulatory and policy-related barriers, proposing increasing awareness and political commitment and improving management of sensitive information for AI use; (3) data-related barriers, suggesting enhancing standardization, collaboration with data networks, managing missing and unstructured data, using analytical and statistical approaches to address bias, using quality assessment tools and quality standards, improving reporting, and developing better conditions for the use of data; and (4) technological barriers, suggesting sustainable development of AI infrastructure.

Abbreviations:

AI= Artficial Intelligence
ATMPs= Advanced Therapy Medicinal Products
CEE= Central and Eastern Europe(an)
EUPATI= Patient Expert Training Programme, https://eupati.eu/
EU= European Union
FAIR= Findable, Accessible, Interoperable and Reusable

HTA= Health Technology Assessment
HTAi PCIG= HTA international Interest Group for Patient and Citizen Involvement in HTA
ME= Middle East
RWD= Real World Data
RWE= Real World Evidence
