# Supplementary material for: Innovating HTA: a call for capacity building and standardization
Source: Int J Technol Assess Health Care. 2026 Jan 28;42(1):e8. doi: 10.1017/S0266462326103456 (PMC12902166; doi:10.1017/S0266462326103456)
Supplement: Delnoij et al. supplementary material 2 — Delnoij et al. supplementary material [file S0266462326103456sup002.docx]

Appendix 2 - Survey HTx Policy and Expert Forum 2024

Which of the following actors do you represent in the HTx Policy and Expert Forum

- Research / academic institutions
- Industry
- National or international governmental bodies
- Patients
- Clinicians
- Payers
- HTA agencies
- Other

Workpackages 1 through 3 of the HTx project were dedicated to methodological innovation of HTA. Workpackages 4 and 5 focussed on implementation of these methods and transferability to Central and Eastern European countries. Several studies done within Work Packages 4 and 5 of the HTx project propose solutions to enhance the use of real world evidence or AI and machine learning in HTA, and to involve stakeholders, notably patient representatives. These solutions include: the use of more advanced personalised HTA methods; capacity building through education and training, and sharing of best practices; standardization and alignment of data definitions, outcome measures and reporting formats; and national and international dialogues, and increased European collaboration. According to you, what are the most important next steps to innovate HTA in the next 5 years:

|  | Very un­im­portant | Un­important | Neutral | Important | Very important |
| --- | --- | --- | --- | --- | --- |
| Further development of methods for analysing real world data | □ | □ | □ | □ | □ |
| Implementation of methods in the EU HTA Regulation | □ | □ | □ | □ | □ |
| Better access to and standardization of real world data | □ | □ | □ | □ | □ |
| Education and training of experts who are doing HTA | □ | □ | □ | □ | □ |
| More and better interaction with stakeholders | □ | □ | □ | □ | □ |

In Europe, training for patient representatives is offered e.g. by EUPATI in the HTA4Patients Project (<https://eupati.eu/hta4patients/>). The need for capacity building within HTA agencies is currently addressed e.g. through tenders of the EU4Health Work Programme 2023, and the EU funded SUSTAIN-HTA project (Support Utilisation of Sustainable and Tailored Innovative Methods for HTA).  In addition, the EHDEN Academy (<https://academy.ehden.eu/>) and the GetReal Academy (<https://getreal-institute.org/education/>) can serve as online educational platforms for a broader group of stakeholders working with new innovative HTA methods.

According to you, how likely is it that capacity building projects or programmes will result in more frequent use of new HTA methods that include the integration of real world evidence for complex health technologies in Europe in the next 5 years?

- Very unlikely
- Unlikely
- Neutral
- Likely
- Very likely

In order to enhance access to standardized real world data high hopes are placed on the European Health Data Space, although much depends on the actual implementation in individual member states. However, the usefulness of HTA methods that integrate the use of real world data for HTA does not only depend on issues of access and interoperability. It is also important that standard sets of patient-relevant outcomes are recorded, including outcomes based on health-related quality of life measures.

According to you, how likely is it that the European Health Data Space will enhance the use of real world evidence as part of HTA methods assessing complex health technologies in Europe in the next 5 years?

- Very unlikely
- Unlikely
- Neutral
- Likely
- Very likely

According to you, how likely is it that as part of the EU HTAR European HTA agencies can enforce the use of standardized sets of outcome measures that include generic measures of health-related quality of life?

- Very unlikely
- Unlikely
- Neutral
- Likely
- Very likely

Collaboration between regulators, HTA agencies and clinical guideline developers as well as stakeholder involvement is suggested in several publications of Work Packages 4 and 5. However, collaboration is time consuming, so for reasons of efficiency HTA agencies may have to prioritize.

If you were in charge of a European HTA agency and for reasons of efficiency you would have to prioritize, which of the following stakeholder groups would you choose to spent most resources on (choose the 3 most important ones)?

- EMA and other regulators
- Other European HTA agencies
- HTA agencies outside the European Union
- Clinical guideline developers
- Patient organisations
- Other
- I don't know / cannot choose
